# Investigating causal relationships between the gut microbiota and allergic diseases: A mendelian randomization study

**DOI:** 10.3389/fgene.2023.1153847

**Published:** 2023-04-12

**Authors:** Yiwei Wang, Tian Liu, Zihao Wan, Lin Wang, Jinpao Hou, Mai Shi, Stephen Kwok Wing Tsui

**Affiliations:** ^1^ School of Biomedical Sciences, Faculty of Medicine, The Chinese University of Hong Kong, Shatin, Hong Kong SAR, China; ^2^ Hong Kong Bioinformatics Centre, The Chinese University of Hong Kong, Shatin, Hong Kong SAR, China; ^3^ Department of Dermatology, The Third Affiliated Hospital off Sun Yat-Sen University, Guangzhou, Hong Kong SAR, China; ^4^ Department of Orthopaedics and Traumatology, Faculty of Medicine, The Chinese University of Hong Kong, Shatin, China; ^5^ Department of Orthopaedics and Limb Reconstruction/Paediatric Orthopaedics, South China Hospital of Shenzhen University, Shenzhen, China; ^6^ Advanced Medical Research Center, Zhengzhou Central Hospital Affiliated to Zhengzhou University, Zhengzhou, China; ^7^ Centre for Microbial Genomics and Proteomics, The Chinese University of Hong Kong, Shatin, Hong Kong SAR, China

**Keywords:** mendelian randomization, allergic disease, genetic varients, genetics, MWAS, gut microbiota (GM)

## Abstract

Observational studies revealed altered gut microbial composition in patients with allergic diseases, which illustrated a strong association between the gut microbiome and the risk of allergies. However, whether such associations reflect causality remains to be well-documented. Two-sample mendelian randomization (2SMR) was performed to estimate the potential causal effect between the gut microbiota and the risk of allergic diseases. 3, 12, and 16 SNPs at the species, genus, and family levels respectively of 15 microbiome features were obtained as the genetic instruments of the exposure dataset from a previous study. GWAS summary data of a total of 17 independent studies related to allergic diseases were collected from the IEU GWAS database for the outcome dataset. Significant causal relationships were obtained between gut microbiome features including *Ruminococcaceae*, *Eggerthella*, *Bifidobacterium*, *Faecalibacterium*, and *Bacteroides* and the risk of allergic diseases. Furthermore, our results also pointed out a number of putative associations between the gut microbiome and allergic diseases. Taken together, this study was the first study using the approach of 2SMR to elucidate the association between gut microbiome and allergic diseases.

## Introduction

Allergic diseases, also known as allergies, including hay fever, allergic rhinitis, asthma, and atopic dermatitis, are the most common chronic immunological diseases and are prevalent in adults and children and persists throughout life (Akdis, 2006). Allergic diseases are mainly caused by IgE-dependent immunological reaction to the allergens and are characterized by the T cell response, including high production of IL-4, IL-5, IL-9, IL-10, and IL-13 and low production of INF-γ (Veldhoen et al., 2008). On the other hand, allergic diseases are genetically predisposed (Aldakheel, 2021). In addition to genetic factors, the gut microbiome has gradually been considered as one of the important risk factors for allergic diseases in recent years due to its influence on the host’s immune response and its interaction with host genetic SNPs (Pascal et al., 2018).

The general term for the microbial community in the human intestine is known as the gut microbiome, disorders of which can cause the occurrence of various diseases such as immune-related, oncologic, neurologic, and metabolic diseases (Gomaa, 2020). Major factors affecting the gut microbiome are divided into three categories: host-extrinsic factors like diet, drugs and lifestyle, host-intrinsic factors such as age, gender, and genetic factors, and environmental factors (Schmidt et al., 2018). The gut microbiome plays a pivotal role in the host’s nutrient metabolism and immunity, which makes it one of the major influencing factors of allergic diseases (Pascal et al., 2018). There are numbers of cross-sectional studies that illustrates there is an altered gut microbiome composition in the patients with allergies. For example, the colonization of *Bacteroides fragilis* was identified to show a positive correlation with the prevalence of asthma during early childhood development. And a relative abundance of *Ruminococcaceae* and *Bifidobacterium* lead to an increase asthma and eczema in infants (Vael et al., 2008; Begley et al., 2018; Berni Canani et al., 2019; Wang et al., 2020; Zhu et al., 2020). However, the causal relationship between gut microbiota composition and allergic disease remains elusive.

The mendelian randomization (MR) model uses genetic variation, the single nucleotide polymorphism (SNP), as the instrumental variable to infer the causal effect size and direction between exposure factors and outcomes (Lawlor et al., 2008). Two-sample mendelian randomization (2SMR) applies the MR methods to estimate the causal effect size of the GWAS summary datasets of two independent studies. This method was wildly used in causal inference detection between the complex diseases such as psychiatric disorders, celiac disease, obesity, T2D, and cardiovascular diseases and specific genera in the host gut microbiome as well as their secretion. Alterations of the relative abundance of *Bifidobacterium* can reduce the risk of ischemic heart disease as well as obesity, and the concentration of the blood low-density lipoprotein. Moreover, changes in the relative abundance of several genera and species, such as *Acidaminococcus*, *Aggregatibacter*, *Blautia*, *Desulfovibrio*, and *Faecalibacterium*, are causes of type 2 diabetes (Yang et al., 2018). Furthermore, Serena et al. suggests that the increase in the content of propionic acid produced by the metabolism of the intestinal flora can also cause an increase in the risk of type 2 diabetes (Sanna et al., 2019). The rich experience of 2SMR and the mature and public GWAS summary databases make the utility of 2SMR on gut microbiome and allergic diseases possible.

In this study, we conducted the two-sample MR analysis to investigate the interplay between host genetics, gut microbiome composition, and allergic diseases including asthma, eczema, hay fever, as well as allergic rhinitis by using GWAS summary datasets from published studies. Our findings provide not only new directions for the treatment and the diagnostic but also valuable insights into early screening of the allergic diseases.

## Materials and methods

Two-sample mendelian randomization analysis was performed using the R package TwoSampleMR (v.0.5.6) (Hemani et al., 2018) and the in-house R scripts used to perform 2SMR analysis and generate figures were available on GitHub (https://github.com/evyforjazz/2SMR). Supplementary Figure S1 illustrated the flowchart of the analysis process.

### Exposure data preparation

Significant SNPs related to the relative abundance of the gut bacteria taxa were selected as the genetic instruments of the exposure data from a public microbiome-GWAS study. Briefly, Goodrich et al. (2016) analyzed the genetic association between 1,300,091 SNPs and 945 taxa of 1126 United Kingdom twin pairs. 307 SNPs were calculated to be correlated with 61 taxa and the summary dataset could be obtained from the Supplementary Table S5 of the original publication. To ensure that the genetic instruments of the exposure data were independent, after acquiring the significant SNPs list, the European (EUR) genotype in the 1,000 human genomes project was used as the reference template for linkage disequilibrium (LD) analysis. The maximum LD R-square value was set to be 0.1 and the distance of searching for LD R-square values was set to be 500 kb. Using a stricter threshold, *p* < 5 × 10^−8^ was the criteria for the selection of the significant SNPs. After performing the step of clumping, 3, 12, and 16 SNPs at the species, genus, and family levels respectively of 15 microbiome features were obtained as the genetic instruments of the exposure dataset for the following two-sample mendelian randomization analysis (Supplementary Table S1).

### Outcome data collection from the IEU GWAS database

IEU GWAS Database (IGD) contains 39,603 GWAS summary studies from 18 batches, which can be obtained and applied to the mendelian randomization analysis using the R package, TwoSampleMR (Abrahamsson et al., 2012). GWAS summary data of a total of 17 independent studies with the trait of allergic asthma, eczema, hay fever, allergic rhinitis, pollen allergy, and medicine or food allergy were collected from IGD (Supplementary Table S2).

### Data harmonization and causal effect evaluation

To make sure the effect of the same SNP of both exposure and outcome data were corresponding to the same allele, the harmonise_data() function of the TwoSampleMR(v0.5.6) package was performed to harmonize the exposure and outcome data. For microbiome features including multiple SNPs, an inverse variance weighted (IVW) was performed to evaluate the causal association. The IVW method is an effective analysis on the assumption that all genetic variations are effective instrumental variables and has a strong causality detection ability (Burgess et al., 2013). For microbiome features containing only 1 SNP, a wild ratio was used to measure the causal effect.

### Sensitivity analysis

MR-Egger regression method was performed to test the horizontal pleiotropy and heterogeneity. Exposure data of the microbiome features containing over 3 SNPs could be used for MR-Egger regression analysis (Bowden et al., 2015). To estimate the causal effect size of each SNP in the microbiome feature allergic diseases, MR_Singlesnp() function was performed. For the microbiome feature with a *p*-value less than 0.05 using the IVW method and passing the heterogeneity analysis and the horizontal pleiotropy analysis, the leave-one-out method was applied for the sensitivity analysis to identify if a single SNP is driving the association.

## Results

### Mendelian randomization results of causal effects between the gut microbiome and atopic dermatitis

**FIGURE 1:**
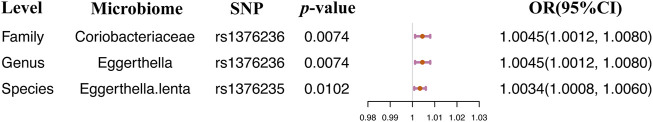
Single SNP analysis between the gut microbiome and AD. Only significant results were displayed.

IVW and WR methods were performed to assess the causal relationship between the abundance of intestinal flora and atopic dermatitis. We found that the causal effect values of the three microbiome features of the same family *Coriobacteriaceae* and eczema were nominally significant (Table 1). MR-Egger regression was tested for heterogeneity and horizontal pleiotropy. And the instrumental variables showed no heterogeneity and pleiotropy, which could be ignored for the causal effect estimation (*p* > 0.05, Supplementary Table S3). The increase of the relative abundance of the *Coriobateriaceae* (*p* = 0.0074) at the family level, *Eggerthella* (*p* = 0.0074) at the genus level, and *Eggerthella lenta* (*p* = 0.0102) at the species level was positively and causally related to the elevating of the risk of atopic dermatitis (Table 1; Figure 1). Only one SNP was related to each significant microbiome feature and the exposure microbiome features *Coriobacteriaceae* at the family level and *Eggerthella* at the genus level shared the same genetic instrument rs1376236. Therefore, the result of the single SNP analysis was the same as the causal estimation analysis results (Figure 1).

**TABLE 1:** Significant 2SMR analysis results between gut microbiome composition and eczema.

Level	Exposure (feature)	IEU GWAS ID	Outcome	Methods	nsnp	*beta*	*se*	*p*-value
Family	*Coriobacteriaceae*	ieu-a-996	Eczema	WR	1	0.0045	0.0017	0.0074
Genus	*Eggerthella*	ieu-a-996		WR	1	0.0045	0.0017	0.0074
Species	*Eggerthella lenta*	ieu-a-996		WR	1	0.0034	0.0013	0.0102

nsnp, number of SNP; WR, wald ratio; ieu-a-996, Eczema.

### Causal effects of gut microbiota on the risk of hay fever, eczema, and allergic rhinitis

**FIGURE 2:**
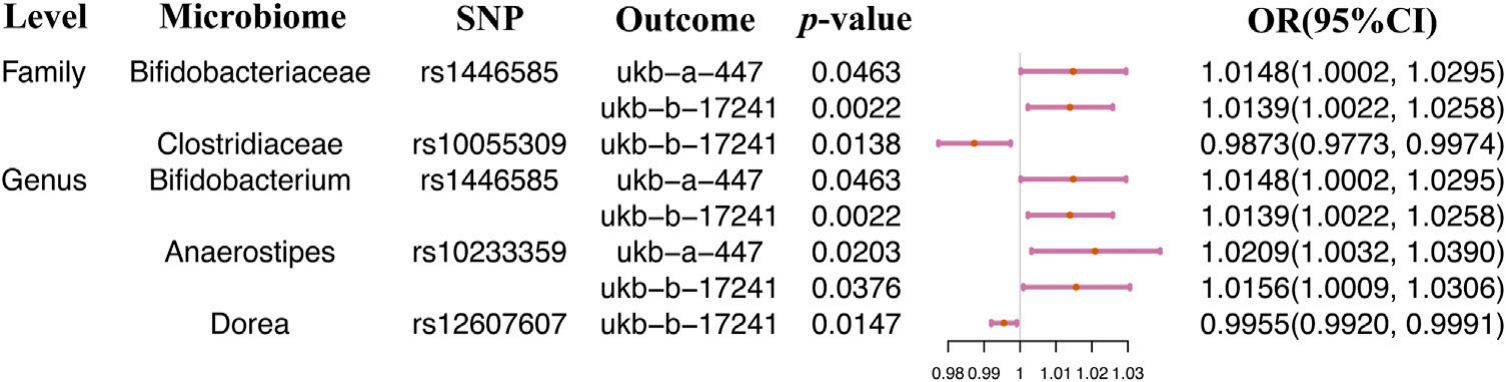
Single SNP analysis between the gut microbiome and eczema, hay fever, and allergic rhinitis.

Significant causal relationships were evaluated between 5 gut microbiome features and hay fever, eczema, or allergic rhinitis (Table 2). The heterogeneity and horizontal pleiotropy could be ignored in the causal estimation between the gut microbiome and allergic diseases (Supplementary Table S4). Our results illustrated that 3 gut microbiome features including *Bifidobacteriaceae* at the family level, *Bifidobacterium* and *Anaerostipes* at the genus level were positively and causally correlated to the risk of hay fever, eczema, or allergic rhinitis. And the 2 microbiome features, *Clostridiaceae* at the family level and *Dorea* at the genus level were negatively and causally related to the risk of hay fever, allergic rhinitis, or eczema (Table 2). Only one SNP was related to each significant microbiome feature. Therefore, the causal effect size and direction of single SNP analysis results was the same as the 2SMR analysis. rs1446585, rs10055309, rs10233359, and rs12604607 were identified to be causally and considerably associated with the risk of hay fever, eczema, and rhinitis (Figure 2).

**TABLE 2:** Significant 2SMR analysis results between gut microbiome composition and hay fever, allergic rhinitis, or eczema.

Level	Exposure (feature)	IEU GWAS ID	Outcome	Methods	nsnp	*beta*	*se*	*p*-value
Family	*Bifidobacteriaceae*	ukb-a-447	Hay fever, allergic rhinitis, or eczema	WR	1	0.0146	0.0073	0.0463
		ukb-b-17241				0.0137	0.0059	0.0201
	*Clostridiaceae*	ukb-b-17241		WR	1	−0.0128	0.0052	0.0140
Genus	*Bifidobacterium*	ukb-a-447		WR	1	0.0147	0.0074	0.0463
		ukb-b-17241				0.0138	0.0059	0.0201
	*Anaerostipes*	ukb-a-447		WR	1	0.0207	0.0089	0.0203
		ukb-b-17241				0.0155	0.0075	0.0376
	*Dorea*	ukb-b-17241		WR	1	−0.0045	0.0018	0.0147

IVW, inverse variance weighted; WR, wald ratio; nsnp, number of SNPs; ukb-a-447, Blood clot DVT, bronchitis, emphysema, asthma, rhinitis, eczema, allergy diagnosed by doctor: Hay fever, allergic rhinitis, or eczema; ukb-b-17241, Blood clot DVT, bronchitis, emphysema, asthma, rhinitis, eczema, allergy diagnosed by doctor: Hay fever, allergic rhinitis, or eczema.

### Seven gut microbiome features were identified causally and significantly related to asthma risk

**FIGURE 3:**
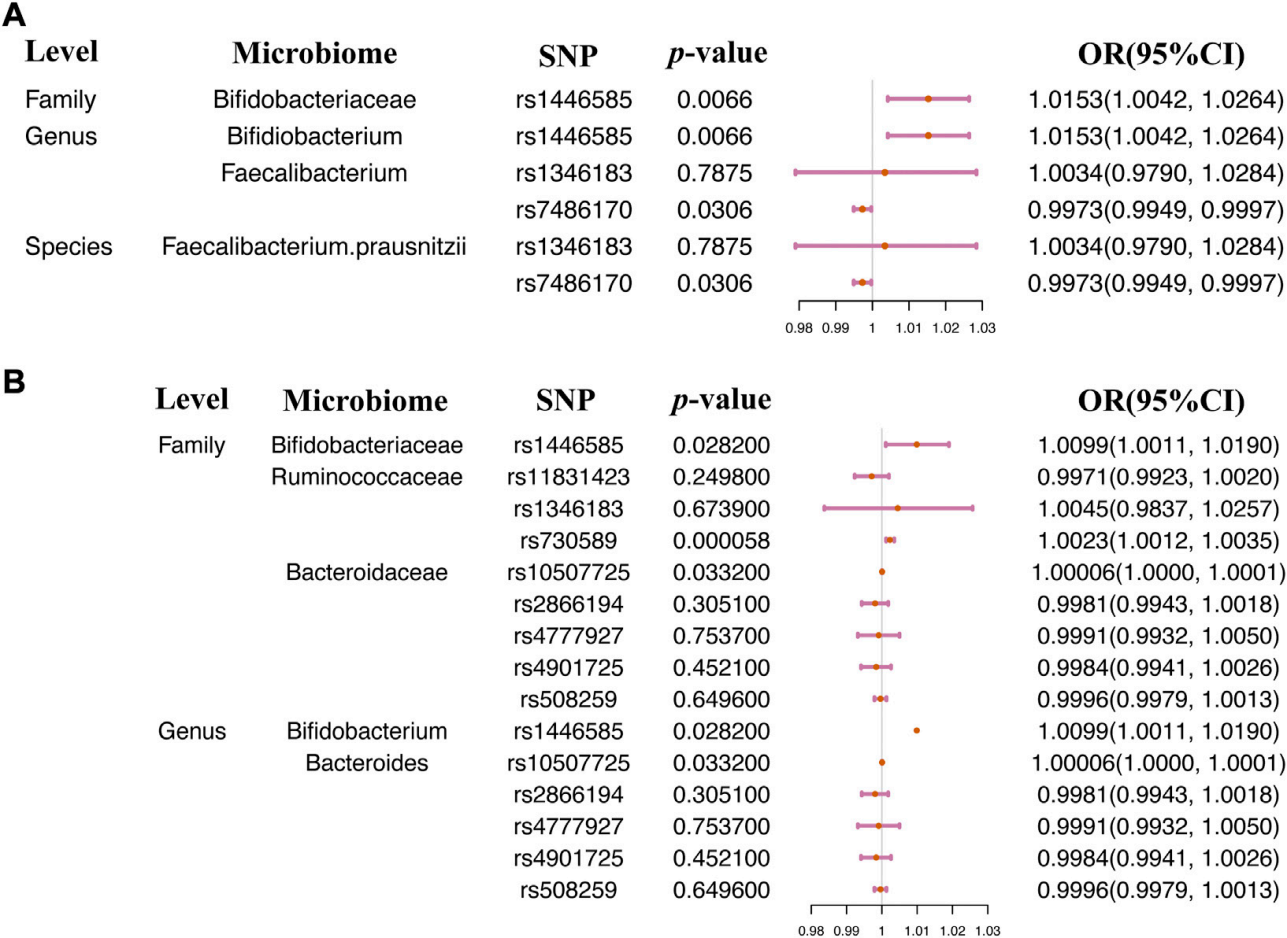
Single SNP analysis between the gut microbiome and asthma. Single SNP analysis was performed on two asthma GWAS summary datasets **(A)** ukb-a-446 and **(B)** ukb-b-20296.

Significant causal relationships were evaluated between 7 gut microbiome features and asthma. The heterogeneity and horizontal pleiotropy could be ignored in the causal estimation between the gut microbiome and asthma (Supplementary Table S4). The increase of the relative abundance of 5 microbiome features, *Bifidobacteriaceae*, *Bacteroidaceae*, and *Ruminococcaceae* at the family level, *Bifidobacterium* and *Bacteroides* at the genus level, and the decrease of the relative abundance of 2 microbiome features, *Faecalibacterium* and *Faecalibacterium prausnitzii*, contribute to the risk of asthma (Table 3). A Leave-one-out analysis was performed on the microbiome features containing over 2SNPs, and the results were consistent with the 2SMR analysis (Supplementary Table S5). Single SNP analysis results were illustrated in Figure 3. 4 SNPs, rs146585 correlated with the microbiome features *Bifidobacteriaceae* at the family level and *Bifidobacterium* at the genus level, rs7486170 associated with the *Faecalibacterium* at the genus level and *F. prausnitzii* at the species level, rs70589 related to *Ruminococcaceae*, and rs10507725 correlated to *Bacteroidaceae* at the family level and *Bacteroides* at the genus level, was detected to demonstrate causal relationships with the risk of asthma (Figure 3).

**TABLE 3:** Significant 2SMR analysis results between gut microbiome composition and asthma.

Level	Exposure (feature)	IEU GWAS ID	Outcome	Methods	nsnp	*beta*	*se*	*p*-value
Family	*Bifidobacteriaceae*	ukb-a-446	Asthma	WR	1	0.0151	0.0055	0.0066
		ukb-b-20296				0.0099	0.0045	0.0282
	*Bacteroidaceae*	ukb-b-20296		IVW	5	5.88E-05	2.82E-05	0.0370
	*Ruminococcaceae*	ukb-b-20296		IVW	3	0.0021	0.0008	0.0113
Genus	*Bifidobacterium*	ukb-a-446		WR	1	0.0151	0.0056	0.0066
		ukb-b-20296				0.0099	0.0045	0.0282
	*Faecalibacterium*	ukb-a-446		IVW	2	−0.0026	0.0012	0.0335
	*Bacteroides*	ukb-b-20296		IVW	5	5.88E-0.5	2.82E-05	0.0370
Species	*F.prausnitzii*	ukb-a-446		IVW	2	−0.0026	0.0012	0.0335

*F. prausnitzii*, *Faecalibacterium prausnitzii*; IVW, inverse variance weighted; WR, wald ratio; ukb-a-446, Blood clot DVT, bronchitis, emphysema, asthma, rhinitis, eczema, allergy diagnosed by doctor: Asthma; ukb-a-20296, Blood clot DVT, bronchitis, emphysema, asthma, rhinitis, eczema, allergy diagnosed by doctor: Asthma.

## Discussion

As far as we know, we are the first to perform the 2SMR approach to assess the potential causal associations between the gut microbiome and allergic diseases including eczema, asthma, hay fever, and allergic rhinitis. We found genetic liability to some gut microbiota causally associated with allergic diseases and some gut microbiota that might be potential risk factors for allergies.

Our results indicated that the enrichment of the *Eggerthella*, belonging to the family *Coriobacteriaceae* and related to rs1376236, as well as its species *E. lenta* correlated with rs1376235 were causally related to atopic dermatitis. Thomas et al. demonstrated that compared with healthy infants, the relative abundance of *Eggerthella* in the colon of the 12-month-old eczema infants raised significantly (Reddel et al., 2019). However, Sofia et al. reported an absolutely opposite result (Abrahamsson et al., 2012). This controversial condition might be attributed to the limited sample size and regional inconsistencies. Moreover, IVs using in causal inference were selected based on the relationship between the intestinal flora and host genetics. And the gut flora was not only affected by host genetic, but also by other factors such as diet, medication, and cultural habits. In addition, *Eggerthella* was detected to be significantly in the gut microbiome of allergic rhinitis patients by comparison with patients with eczema and measles (Su et al., 2021). Furthermore, *E. lenta* under the genera *Eggerthella* was shown to be highly enriched in the gut flora of patients with asthma and rheumatoid arthritis, comparing to the normal population (Chen et al., 2016; Wang et al., 2018). Additionally, the colonization of the *E. lenta* was evidenced to induce the activation of the Th17, leading to colitis (Alexander et al., 2019).

We also found that *Ruminococcaceae* at the family level, and *Bifidobacterium* and *Bacteroides* at the genus level were positively associated with the risk of asthma. Previous studies revealed that a higher abundance of *Ruminococcaceae* was colonized in the intestinal flora of infants with food allergies and atopic eczema by comparison with healthy infants (Berni Canani et al., 2019; Zhu et al., 2020). Besides, the results of a tracking study by Carl et al. on the gut microbiome of 117 infants supported our causal inference results regarding *Bacteroides* and the risk of asthma. Carl et al. found that the colonization of *B. fragilis* was positively correlated with the prevalence of asthma during early development. They also put forward a proposal that the enrichment of the *B. fragilis* in early life played a role as a potential biomarker of possible asthma (Vael et al., 2008). In addition, Begley et al. (2018) demonstrated that compared with normal children, the relative abundance of *Bifidobacterium* declined significantly in the gut bacteria community of children at high risk of asthma. Notably, certain strains of *Bifidobacterium* had been illustrated to alleviate asthma *via* inhibiting Th1/Th2 activation (Wang et al., 2020). The inconsistency between our causal estimation and the results of clinical trials as stated above might be traceable to the following reasons. First, as mentioned above, our causal evaluation was based on the host genetic variations. Both gut microbiome and human diseases were not only influenced by host inheritance but also affected by other factors such as diet and lifestyle. Second, pleiotropic testing and sensitivity analysis could not be performed because only one SNP, rs1446585, was selected as the exposure IV of *Bifidobacterium*. Thus, we failed to exclude the impact of the horizontal pleiotropy on causal inference between *Bifidobacterium* and asthma.

Conversely, we evidenced that the increase of *F. prausnitzii* potentially resulted in a lessening of the risk of asthma, which supported the findings of the longitudinal study conducted by Galazzo et al. (2020) (Alexander et al., 2019). In children who developed eczema and asthma, the deficiency of *Faecalibacterium* was detected in their intestinal microbiome during infancy. Similar conclusions were reached by Arrieta et al. (2015) and Stokholm et al. (2018) using sequencing approaches.

Interestingly, we found that the significant causal relationships between *Clostridiaceae* at the family level and *Dorea* at the genus level and the risk of hay fever, allergic rhinitis, or eczema allergic could not be replicated in two similar outcome studies. The same inconsistent results were also seen when assessing the causal association between *Bacteroidaceae* and *Ruminococcaceae* at the family level, *Bacteroides and Faecalibacterium* at the genus level, and asthma risk. First, this may be due to the limited number of SNPs included in this study, resulting in insufficient statistical significance to identify associations. Second, the gut microbiome composition is more affected by environmental factors. Genetic differences have a minimal impact on given exposure, which could just explain a small proportion of the alteration of the gut microbiome. So, in some cases, our analysis might have had insufficient power to detect a causal relationship.

The present study had several limitations. First, the IVs selected in this study were all correlated to the relative abundance of the individual bacteria in the gut microbiome, but the IVs related to the functional metabolic pathways of the gut flora were lacking. The metabolites of the intestinal microbiome might be directly related to human diseases and illustrate a more intuitive causal association with the diseases. Besides, the relatively small sample size of the microbiome-GWAS study (*n* = 1,126 United Kingdom twins) used in this study might lead to some bacteria that were found to be potentially causally related to allergic disease in one study, but could not be identified repeatedly in another study, which reduced the confidence of the true causal relationship. Moreover, most of the bacteria taxa analyzed in this study were at the genus level, and future analysis at the level of species and strains will improve the accuracy of causality. In addition, our study only used GWAS summary data to complete the analysis of 2SMR and ignored the bacterial composition across samples. In the future research, when the compositionality of the gut microbiome is addressed, GutBalance and DisBalance can be used to accurately predict the microbial biomarkers of the allergic diseases (Yang et al., 2021; Yang and Zou, 2021).

## Conclusion

Taken together, this study supports the potential causal effects of gut microbial composition on allergies, including eczema, asthma, hay fever, and allergic rhinitis. Our research puts forward the hypothesis that *Ruminococcaceae*, *Eggerthella*, *Bifidobacterium*, *Faecalibacterium*, and *Bacteroides* might be potential risk factors for allergic diseases. In summary, our work can shed light on the comprehensive interactions between the host and the gut flora in allergic patients and provide new directions as well as novel strategies for the treatment, diagnosis, and early screening of atopic eczema, asthma, and related allergic diseases.

## Data Availability

The datasets presented in this study can be found in online repositories. The names of the repository/repositories and accession number(s) can be found in the article/Supplementary Material.
