# Supplementary material for: Investigating causal relationships between the gut microbiota and allergic diseases: A mendelian randomization study
Source: Front Genet. 2023 Apr 12;14:1153847. doi: 10.3389/fgene.2023.1153847 (PMC10130909; doi:10.3389/fgene.2023.1153847)
Supplement: Supplementary file 1 [file DataSheet2.PDF]

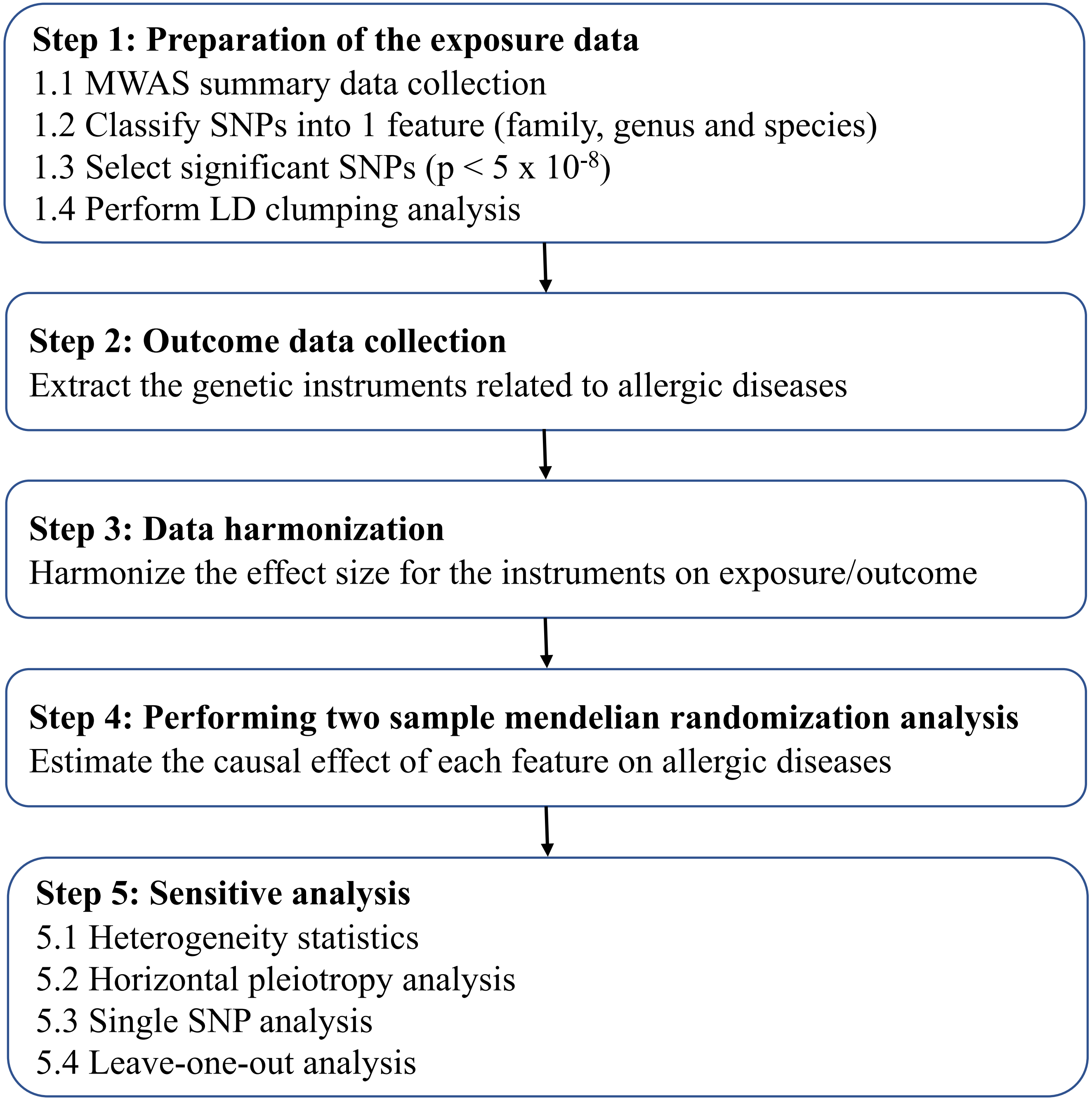
Step 1: Preparation of the exposure data
1.1 MWAS summary data collection
1.2 Classify SNPs into 1 feature (family, genus and species)
1.3 Select significant SNPs ($p < 5 \times 10^{-8}$)
1.4 Perform LD clumping analysis
Step 2: Outcome data collection
Extract the genetic instruments related to allergic diseases
Step 3: Data harmonization
Harmonize the effect size for the instruments on exposure/outcome
Step 4: Performing two sample mendelian randomization analysis
Estimate the causal effect of each feature on allergic diseases
Step 5: Sensitive analysis
5.1 Heterogeneity statistics
5.2 Horizontal pleiotropy analysis
5.3 Single SNP analysis
5.4 Leave-one-out analysis
