# Supplementary figures and images for: Investigating causal relationships between the gut microbiota and allergic diseases: A mendelian randomization study
Source: Front Genet. 2023 Apr 12;14:1153847. doi: 10.3389/fgene.2023.1153847 (PMC10130909; doi:10.3389/fgene.2023.1153847)

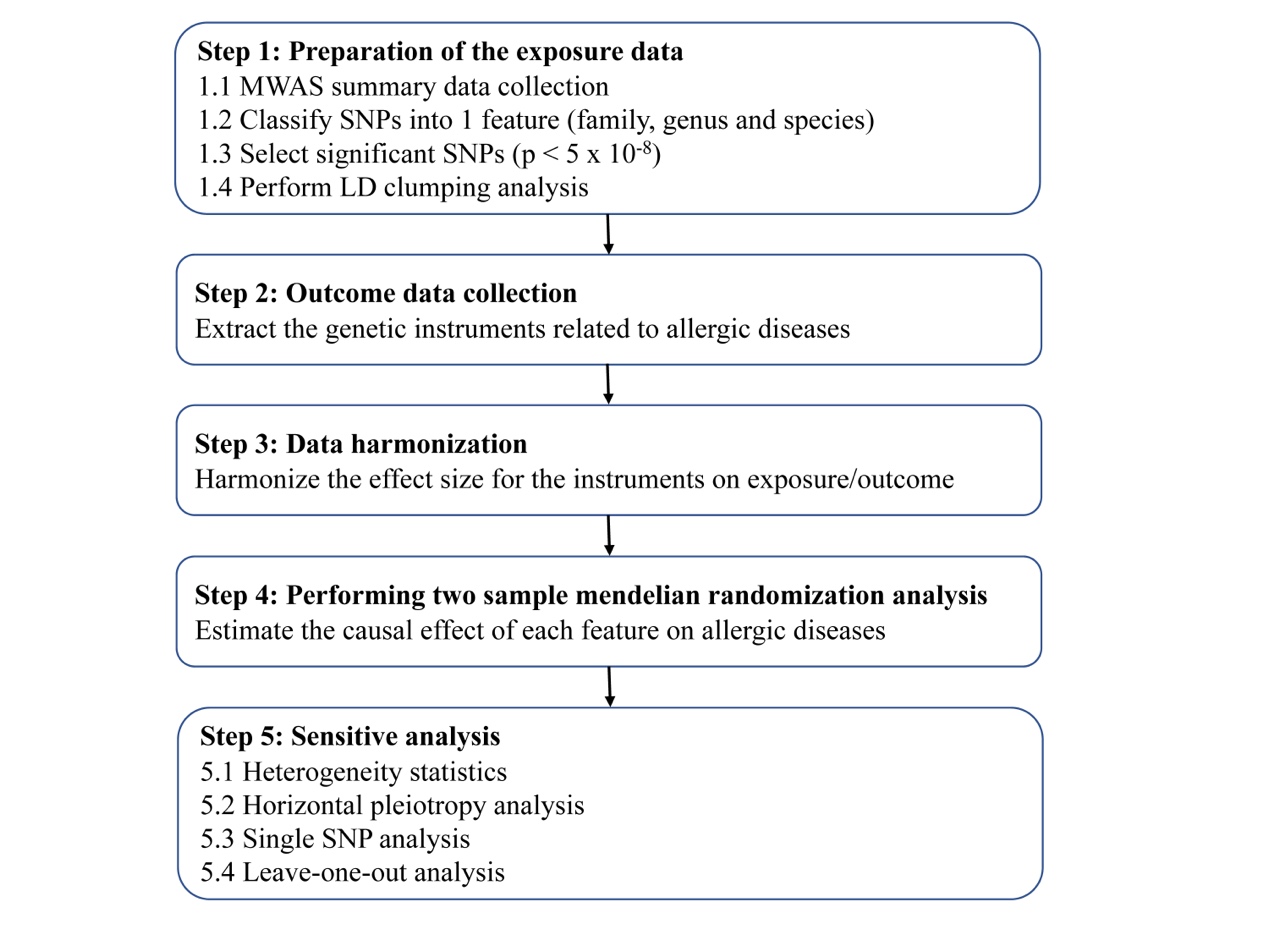


**Figure S1** Flowchart of the 2SMR analysis process.

Supplement: Supplementary file 2 [file Table1.DOCX]
