# Supplementary material for: Investigating causal relationships between the gut microbiota and allergic diseases: A mendelian randomization study
Source: Front Genet. 2023 Apr 12;14:1153847. doi: 10.3389/fgene.2023.1153847 (PMC10130909; doi:10.3389/fgene.2023.1153847)
Supplement: Supplementary file 3 [file Table2.DOCX]

**Supplementary tables**

**Table S1** GWAS summary data of each microbiota feature from the TwinsUK registry after clumping at *P*-value<5.0×10^-8^

| **Level** | **Feature** | **SNP** | **A1** | **A2** | **EAF** | ***beta*** | ***se*** | ***p*-value** |
| --- | --- | --- | --- | --- | --- | --- | --- | --- |
| Family | *Bacteroidaceae* | rs4777927 | T | C | 0.336 | 0.236 | 0.0417 | 1.67×10^-8^ |
|  |  | rs4901725 | C | T | 0.355 | -0.316 | 0.0564 | 2.43×10^-8^ |
|  |  | rs508259 | C | T | 0.095 | -1.27 | 0.228 | 3.24×10^-8^ |
|  |  | rs2866194 | G | A | 0.288 | -0.38 | 0.0692 | 4.45×10^-8^ |
|  |  | rs10507725 | A | C | 0.089 | 43.2 | 7.88 | 4.71×10^-8^ |
|  | *Bifidobacteriaceae* | rs1446585 | G | A | 0.265 | 0.168 | 0.0305 | 3.75×10^-8^ |
|  | *Clostridiaceae* | rs10055309 | C | T | 0.059 | 0.353 | 0.0617 | 1.20×10^-8^ |
|  | *Coriobacteriaceae* | rs1376236 | C | A | 0.085 | 16.3 | 2.87 | 1.37×10^-8^ |
|  | *Lachnospiraceae* | rs10233359 | A | G | 0.075 | -0.211 | 0.0359 | 4.94×10^-8^ |
|  |  | rs2293702 | T | C | 0.477 | 0.078 | 0.0137 | 1.41×10^-8^ |
|  |  | rs11880147 | G | A | 0.197 | 5.21 | 0.933 | 2.59×10^-8^ |
|  |  | rs12607607 | T | C | 0.169 | -0.63 | 0.114 | 3.69×10^-8^ |
|  |  | rs498018 | T | G | 0.39 | 3.38 | 0.614 | 3.92×10^-8^ |
|  | *Ruminococcaceae* | rs730589 | T | G | 0.337 | -1.2 | 0.21 | 1.37×10^-8^ |
|  |  | rs11831423 | A | C | 0.126 | 0.396 | 0.0706 | 2.33×10^-8^ |
|  |  | rs1346183 | C | T | 0.105 | -0.0983 | 0.0177 | 3.21×10^-8^ |
| Genus | *Anaerostipes* | rs10233359 | A | G | 0.075 | -0.211 | 0.0359 | 4.94×10^-8^ |
|  | *Bacteroides* | rs4777927 | T | C | 0.336 | 0.236 | 0.0417 | 1.67×10^-8^ |
|  |  | rs4901725 | C | T | 0.355 | -0.316 | 0.0564 | 2.43×10^-8^ |
|  |  | rs508259 | C | T | 0.095 | -1.27 | 0.228 | 3.24×10^-8^ |
|  |  | rs2866194 | G | A | 0.288 | -0.38 | 0.0692 | 4.45×10^-8^ |
|  |  | rs10507725 | A | C | 0.089 | 43.2 | 7.88 | 4.71×10^-8^ |
|  | *Bifidobacterium* | rs1446585 | G | A | 0.265 | 0.167 | 0.0303 | 3.89×10^-8^ |
|  | *Coprococcus* | rs2293702 | T | C | 0.477 | 0.078 | 0.0137 | 1.41×10^-8^ |
|  | *Dorea* | rs12607607 | T | C | 0.169 | -0.63 | 0.114 | 3.69×10^-8^ |
|  | *Eggerthella* | rs1376236 | C | A | 0.085 | 16.3 | 2.87 | 1.37×10^-8^ |
|  | *Faecalibacterium* | rs1346183 | C | T | 0.105 | -0.0983 | 0.0177 | 3.21×10^-8^ |
|  |  | rs7486170 | A | G | 0.124 | 0.935 | 0.17 | 4.38×10^-8^ |
| Species | *Eggerthella. lenta* | rs1376235 | G | C | 0.083 | 21.1 | 3.81 | 3.55×10^-8^ |
|  | *Faecalibacterium. prausnitzii* | rs1346183 | C | T | 0.105 | -0.0983 | 0.0177 | 3.21×10^-8^ |
|  |  | rs7486170 | A | G | 0.124 | 0.935 | 0.17 | 4.38×10^-8^ |

**Table S2** GWAS summary datasets of outcome data of allergic diseases in the IEU GWAS database.

| No. | IEU GWAS ID | Trait | Sample size | Number of SNPs |
| --- | --- | --- | --- | --- |
| 1 | ukb-b-5911 | Diagnoses - secondary ICD10: Z88.0 Personal history of allergy to penicillin | 463,010 | 9,851,867 |
| 2 | ukb-b-9841 | Diagnoses - secondary ICD10: Z88.1 Personal history of allergy to other antibiotic agents | 463,010 | 9,851,867 |
| 3 | ukb-b-4601 | Diagnoses - secondary ICD10: Z88.8 Personal history of allergy to other drugs, medicaments and biological substances | 463,010 | 9,851,867 |
| 4 | ukb-b-16702 | Diagnoses - secondary ICD10: Z91.0 Personal history of allergy, other than to drugs and biological substances | 463,010 | 9,851,867 |
| 5 | ukb-b-10351 | Non-cancer illness code, self-reported: allergy or anaphylactic reaction to drug | 462,933 | 9,851,867 |
| 6 | ukb-b-18787 | Non-cancer illness code, self-reported: allergy or anaphylactic reaction to food | 462,933 | 9,851,867 |
| 7 | ukb-b-9039 | Non-cancer illness code, self-reported: allergy/hypersensitivity/anaphylaxis | 462,933 | 9,851,867 |
| 8 | ukb-b-20296 | Blood clot, DVT, bronchitis, emphysema, asthma, rhinitis, eczema, allergy diagnosed by doctor: Asthma | 462,013 | 9,851,857 |
| 9 | ukb-b-16207 | Blood clot, DVT, bronchitis, emphysema, asthma, rhinitis, eczema, allergy diagnosed by doctor: Emphysema/chronic bronchitis | 462,013 | 9,851,857 |
| 10 | ukb-b-17241 | Blood clot, DVT, bronchitis, emphysema, asthma, rhinitis, eczema, allergy diagnosed by doctor: Hayfever, allergic rhinitis or eczema | 462,013 | 9,851,857 |
| 11 | ukb-a-93 | Non-cancer illness code self-reported: allergy or anaphylactic reaction to drug | 337,159 | 10,894,596 |
| 12 | ukb-a-446 | Blood clot DVT bronchitis emphysema asthma rhinitis eczema allergy diagnosed by doctor: Asthma | 337,159 | 10,894,596 |
| 13 | ukb-a-444 | Blood clot DVT bronchitis emphysema asthma rhinitis eczema allergy diagnosed by doctor: Emphysema/chronic bronchitis | 337,159 | 10,894,596 |
| 14 | ukb-a-447 | Blood clot DVT bronchitis emphysema asthma rhinitis eczema allergy diagnosed by doctor: Hay fever allergic rhinitis or eczema | 337,159 | 10,894,596 |
| 15 | finn-a-CHILDHOOD_ALLERGY | Childhood allergy (age < 16) | - | 16,152,119 |
| 16 | finn-b-POLLENALLERGY | Pollen allergy | 217,436 | 16,380,460 |
| 17 | ieu-a-996 | Eczema | 40,835 | 11,059,641 |

**Table S3** Heterogeneity analysis and horizontal pleiotropy analysis of gut microbiome on eczema.

| Exposure (Feature) | Outcome | Horizontal pleiotropy analysis | | Heterogeneity analysis | |
| --- | --- | --- | --- | --- | --- |
|  |  | egger_intercapt | p-value | Q | Q_pval |
| *Lachnospiraceae* | ieu-a-996 | -0.0093 | 0.6668 | 7.09 | 0.0691 |
| *Ruminococcaceae* | ieu-a-996 | -0.0017 | 0.9591 | 1.27 | 0.2591 |
| *Bacteroidaceae* | ieu-a-996 | 0.0015 | 0.8917 | 3.40 | 0.3339 |
| *Bacteroides* | ieu-a-996 | 0.0015 | 0.8917 | 3.40 | 0.3339 |

ieu-a-996: Eczema

**Table S4** Heterogeneity analysis and horizontal pleiotropy analysis of gut microbiome on allergic diseases.

| Exposure (Feature) | Outcome | Horizontal pleiotropy analysis | | Heterogeneity analysis | |
| --- | --- | --- | --- | --- | --- |
|  |  | egger_intercapt | p-value | Q | Q_pval |
| *Lachnospiraceae* | ukb-a-446 | -0.0001 | 0.8390 | 2.4278 | 0.4888 |
|  | ukb-a-447 | 3.17E-04 | 0.8616 | 13.1307 | 0.0004 |
|  | ukb-b-20296 | -0.0005 | 0.5406 | 5.0780 | 0.1662 |
|  | ukb-b-17241 | -1.03E-05 | 0.9943 | 11.4578 | 0.0095 |
| *Ruminococcaceae* | ukb-a-446 | -0.0017 | 0.6182 | 5.3706 | 0.0205 |
|  | ukb-a-447 | -6.09E-04 | 0.7430 | 0.4196 | 0.5171 |
|  | ukb-b-20296 | -0.0010 | 0.6554 | 3.0832 | 0.0791 |
|  | ukb-b-17241 | 7.15E-04 | 0.6659 | 1.0455 | 0.3065 |
| *Bacteroidaceae* | ukb-a-446 | -0.0003 | 0.5147 | 0.5816 | 0.9001 |
|  | ukb-a-447 | 3.75E-06 | 0.9954 | 1.1904 | 0.7553 |
|  | ukb-b-20296 | -0.0005 | 0.2691 | 0.2800 | 0.9367 |
|  | ukb-b-17241 | -2.39E-07 | 0.9997 | 0.1598 | 0.9838 |
| *Bacteroides* | ukb-a-446 | -0.0003 | 0.5142 | 0.5816 | 0.9001 |
|  | ukb-a-447 | 3.75E-06 | 0.9954 | 1.1904 | 0.7533 |
|  | ukb-b-20296 | -0.0005 | 0.2691 | 0.2800 | 0.9637 |
|  | ukb-b-17241 | -2.39E-07 | 0.9997 | 0.1598 | 0.9838 |

ukb-a-446: Blood clot DVT bronchitis, emphysema, asthma, rhinitis, eczema, allergy diagnosed by doctor: Asthma; IGD: ukb-a-20296: Blood clot DVT bronchitis, emphysema, asthma, rhinitis, eczema, allergy diagnosed by doctor: Asthma. ukb-a-447: Blood clot DVT bronchitis, emphysema, asthma, rhinitis, eczema, allergy diagnosed by doctor: Hay fever, allergic rhinitis, or eczema; ukb-b-17241: Blood clot DVT bronchitis, emphysema, asthma, rhinitis, eczema, allergy diagnosed by doctor: Hay fever, allergic rhinitis, or eczema.

**Table S5** Leave-one-out analysis results between gut microbiome composition and asthma.

| Level | Exposure (Feature) | Outcome | Methods | nsnp | *beta* | *se* | *p*-value |
| --- | --- | --- | --- | --- | --- | --- | --- |
| Family | *Bacteroidaceae* | ukb-b-20296 | IVW | 5 | 5.88E-05 | 2.82E-05 | 0.0370 |
|  | *Ruminococcaceae* | ukb-b-20296 | IVW | 3 | 0.0021 | 0.0008 | 0.0113 |
| Genus | *Faecalibacterium* | ukb-a-446 | IVW | 2 | -0.0026 | 0.0012 | 0.0335 |
|  | *Bacteroides* | ukb-b-20296 | IVW | 5 | 5.88E-05 | 2.82E-05 | 0.0370 |
| Species | *F.prausnitzii* | ukb-a-446 | IVW | 2 | -0.0026 | 0.0012 | 0.0335 |

*F.prausnitzii*: *Faecalibacterium.prausnitzii*; IVW: Inverse Variance Weighted; ukb-a-446: Blood clot DVT bronchitis, emphysema, asthma, rhinitis, eczema, allergy diagnosed by doctor: Asthma; IGD: ukb-a-20296: Blood clot DVT bronchitis, emphysema, asthma, rhinitis, eczema, allergy diagnosed by doctor: Asthma.
