# Supplementary material for: Investigating causal relationships between the gut microbiota and allergic diseases: A mendelian randomization study
Source: Front Genet. 2023 Apr 12;14:1153847. doi: 10.3389/fgene.2023.1153847 (PMC10130909; doi:10.3389/fgene.2023.1153847)
Supplement: Supplementary file 4 [file DataSheet1.PDF]

Supplementary tables

Table S1 GWAS summary data of each microbiota feature from the TwinsUK registry after clumping at *P*-value<5.0×10^{-8}

Level	Feature	SNP	A1	A2	EAF	*beta*	*se*	*p*-value
Family	*Bacteroidaceae*	rs4777927	T	C	0.336	0.236	0.0417	1.67×10^{-8}
		rs4901725	C	T	0.355	-0.316	0.0564	2.43×10^{-8}
		rs508259	C	T	0.095	-1.27	0.228	3.24×10^{-8}
		rs2866194	G	A	0.288	-0.38	0.0692	4.45×10^{-8}
		rs10507725	A	C	0.089	43.2	7.88	4.71×10^{-8}
	Bifidobacteriaceae	rs1446585	G	A	0.265	0.168	0.0305	3.75×10^{-8}
	Clostridiaceae	rs10055309	C	T	0.059	0.353	0.0617	1.20×10^{-8}
	Coriobacteriaceae	rs1376236	C	A	0.085	16.3	2.87	1.37×10^{-8}
	Lachnospiraceae	rs10233359	A	G	0.075	-0.211	0.0359	4.94×10^{-8}
		rs2293702	T	C	0.477	0.078	0.0137	1.41×10^{-8}
		rs11880147	G	A	0.197	5.21	0.933	2.59×10^{-8}
		rs12607607	T	C	0.169	-0.63	0.114	3.69×10^{-8}
		rs498018	T	G	0.39	3.38	0.614	3.92×10^{-8}
	Ruminococcaceae	rs730589	T	G	0.337	-1.2	0.21	1.37×10^{-8}
		rs11831423	A	C	0.126	0.396	0.0706	2.33×10^{-8}
		rs1346183	C	T	0.105	-0.0983	0.0177	3.21×10^{-8}
Genus	*Anaerostipes*	rs10233359	A	G	0.075	-0.211	0.0359	4.94×10^{-8}
	Bacteroides	rs4777927	T	C	0.336	0.236	0.0417	1.67×10^{-8}
		rs4901725	C	T	0.355	-0.316	0.0564	2.43×10^{-8}
		rs508259	C	T	0.095	-1.27	0.228	3.24×10^{-8}
		rs2866194	G	A	0.288	-0.38	0.0692	4.45×10^{-8}
		rs10507725	A	C	0.089	43.2	7.88	4.71×10^{-8}
	Bifidobacterium	rs1446585	G	A	0.265	0.167	0.0303	3.89×10^{-8}
	Coprococcus	rs2293702	T	C	0.477	0.078	0.0137	1.41×10^{-8}
	Dorea	rs12607607	T	C	0.169	-0.63	0.114	3.69×10^{-8}
	Eggerthella	rs1376236	C	A	0.085	16.3	2.87	1.37×10^{-8}
	Faecalibacterium	rs1346183	C	T	0.105	-0.0983	0.0177	3.21×10^{-8}
		rs7486170	A	G	0.124	0.935	0.17	4.38×10^{-8}
Species	*Eggerthella. lenta*	rs1376235	G	C	0.083	21.1	3.81	3.55×10^{-8}
	Faecalibacterium.	rs1346183	C	T	0.105	-0.0983	0.0177	3.21×10^{-8}
	prausnitzii	rs7486170	A	G	0.124	0.935	0.17	4.38×10^{-8}

Table S2 GWAS summary datasets of outcome data of allergic diseases in the IEU GWAS database.

No.	IEU GWAS ID	Trait	Sample size	Number of SNPs
1	ukb-b-5911	Diagnoses - secondary ICD10: Z88.0 Personal history of allergy to penicillin	463,010	9,851,867
2	ukb-b-9841	Diagnoses - secondary ICD10: Z88.1 Personal history of allergy to other antibiotic agents	463,010	9,851,867
3	ukb-b-4601	Diagnoses - secondary ICD10: Z88.8 Personal history of allergy to other drugs, medicaments and biological substances	463,010	9,851,867
4	ukb-b-16702	Diagnoses - secondary ICD10: Z91.0 Personal history of allergy, other than to drugs and biological substances	463,010	9,851,867
5	ukb-b-10351	Non-cancer illness code, self-reported: allergy or anaphylactic reaction to drug	462,933	9,851,867
6	ukb-b-18787	Non-cancer illness code, self-reported: allergy or anaphylactic reaction to food	462,933	9,851,867
7	ukb-b-9039	Non-cancer illness code, self-reported: allergy/hypersensitivity/anaphylaxis	462,933	9,851,867
8	ukb-b-20296	Blood clot, DVT, bronchitis, emphysema, asthma, rhinitis, eczema, allergy diagnosed by doctor: Asthma	462,013	9,851,857
9	ukb-b-16207	Blood clot, DVT, bronchitis, emphysema, asthma, rhinitis, eczema, allergy diagnosed by doctor: Emphysema/chronic bronchitis	462,013	9,851,857
10	ukb-b-17241	Blood clot, DVT, bronchitis, emphysema, asthma, rhinitis, eczema, allergy diagnosed by doctor: Hayfever, allergic rhinitis or eczema	462,013	9,851,857
11	ukb-a-93	Non-cancer illness code self-reported: allergy or anaphylactic reaction to drug	337,159	10,894,596

12	ukb-a-446	Blood clot DVT bronchitis emphysema asthma rhinitis eczema allergy diagnosed by doctor: Asthma	337,159	10,894,596
13	ukb-a-444	Blood clot DVT bronchitis emphysema asthma rhinitis eczema allergy diagnosed by doctor: Emphysema/chronic bronchitis	337,159	10,894,596
14	ukb-a-447	Blood clot DVT bronchitis emphysema asthma rhinitis eczema allergy diagnosed by doctor: Hay fever allergic rhinitis or eczema	337,159	10,894,596
15	finn-a-CHILDHOOD_ALLERGY	Childhood allergy (age < 16)	-	16,152,119
16	finn-b-POLLENALLERGY	Pollen allergy	217,436	16,380,460
17	ieu-a-996	Eczema	40,835	11,059,641

Table S3 Heterogeneity analysis and horizontal pleiotropy analysis of gut microbiome on eczema.

Exposure (Feature)	Outcome	Horizontal pleiotropy analysis		Heterogeneity analysis	
		egger_intercapt	p-value	Q	Q_pval
Lachnospiraceae	ieu-a-996	-0.0093	0.6668	7.09	0.0691
Ruminococcaceae	ieu-a-996	-0.0017	0.9591	1.27	0.2591
Bacteroidaceae	ieu-a-996	0.0015	0.8917	3.40	0.3339
Bacteroides	ieu-a-996	0.0015	0.8917	3.40	0.3339

ieu-a-996: Eczema

Table S4 Heterogeneity analysis and horizontal pleiotropy analysis of gut microbiome on allergic diseases.

Exposure (Feature)	Outcome	Horizontal pleiotropy analysis		Heterogeneity analysis	
		egger_intercapt	p-value	Q	Q_pval
Lachnospiraceae	ukb-a-446	-0.0001	0.8390	2.4278	0.4888
	ukb-a-447	3.17E-04	0.8616	13.1307	0.0004
	ukb-b-20296	-0.0005	0.5406	5.0780	0.1662
	ukb-b-17241	-1.03E-05	0.9943	11.4578	0.0095
Ruminococcaceae	ukb-a-446	-0.0017	0.6182	5.3706	0.0205
	ukb-a-447	-6.09E-04	0.7430	0.4196	0.5171
	ukb-b-20296	-0.0010	0.6554	3.0832	0.0791
	ukb-b-17241	7.15E-04	0.6659	1.0455	0.3065
Bacteroidaceae	ukb-a-446	-0.0003	0.5147	0.5816	0.9001
	ukb-a-447	3.75E-06	0.9954	1.1904	0.7553
	ukb-b-20296	-0.0005	0.2691	0.2800	0.9367
	ukb-b-17241	-2.39E-07	0.9997	0.1598	0.9838
Bacteroides	ukb-a-446	-0.0003	0.5142	0.5816	0.9001
	ukb-a-447	3.75E-06	0.9954	1.1904	0.7533
	ukb-b-20296	-0.0005	0.2691	0.2800	0.9637
	ukb-b-17241	-2.39E-07	0.9997	0.1598	0.9838

ukb-a-446: Blood clot DVT bronchitis, emphysema, asthma, rhinitis, eczema, allergy diagnosed by doctor: Asthma; IGD: ukb-a-20296: Blood clot DVT bronchitis, emphysema, asthma, rhinitis, eczema, allergy diagnosed by doctor: Asthma. ukb-a-447: Blood clot DVT bronchitis, emphysema, asthma, rhinitis, eczema, allergy diagnosed by doctor: Hay fever, allergic rhinitis, or eczema; ukb-b-17241: Blood clot DVT bronchitis, emphysema, asthma, rhinitis, eczema, allergy diagnosed by doctor: Hay fever, allergic rhinitis, or eczema.

Table S5 Leave-one-out analysis results between gut microbiome composition and asthma.

Level	Exposure (Feature)	Outcome	Methods	nsnp	*beta*	*se*	*p*-value
Family	*Bacteroidaceae*	ukb-b-20296	IVW	5	5.88E-05	2.82E-05	0.0370
	Ruminococcaceae	ukb-b-20296	IVW	3	0.0021	0.0008	0.0113
Genus	*Faecalibacterium*	ukb-a-446	IVW	2	-0.0026	0.0012	0.0335
	Bacteroides	ukb-b-20296	IVW	5	5.88E-05	2.82E-05	0.0370
Species	*F.prausnitzii*	ukb-a-446	IVW	2	-0.0026	0.0012	0.0335

F.prausnitzii: *Faecalibacterium.prausnitzii*; IVW: Inverse Variance Weighted; ukb-a-446: Blood clot DVT bronchitis, emphysema, asthma, rhinitis, eczema, allergy diagnosed by doctor: Asthma; IGD: ukb-a-20296: Blood clot DVT bronchitis, emphysema, asthma, rhinitis, eczema, allergy diagnosed by doctor: Asthma.
